# A catalogue with semantic annotations makes multilabel datasets FAIR

**DOI:** 10.1038/s41598-022-11316-3

**Published:** 2022-05-04

**Authors:** Ana Kostovska, Jasmin Bogatinovski, Sašo Džeroski, Dragi Kocev, Panče Panov

**Affiliations:** 1 Department of Knowledge Technologies, Jožef Stefan Institute, Ljubljana, Slovenia; 2 Jožef Stefan International Postgraduate School, Ljubljana, Slovenia; 3 Department of Distributed and Operating Systems, Technical University Berlin, Berlin, Germany; 4 Bias Variance Labs, d.o.o., Ljubljana, Slovenia

**Keywords:** Computer science, Scientific data, Information technology

## Abstract

Multilabel classification (MLC) is a machine learning task where the goal is to learn to label an example with multiple labels simultaneously. It receives increasing interest from the machine learning community, as evidenced by the increasing number of papers and methods that appear in the literature. Hence, ensuring proper, correct, robust, and trustworthy benchmarking is of utmost importance for the further development of the field. We believe that this can be achieved by adhering to the recently emerged data management standards, such as the FAIR (Findable, Accessible, Interoperable, and Reusable) and TRUST (Transparency, Responsibility, User focus, Sustainability, and Technology) principles. We introduce an ontology-based online catalogue of MLC datasets originating from various application domains following these principles. The catalogue extensively describes many MLC datasets with comprehensible meta-features, MLC-specific semantic descriptions, and different data provenance information. The MLC data catalogue is available at: http://semantichub.ijs.si/MLCdatasets.

## Introduction

Supervised learning is a machine learning task focused on learning models that provide the value of a selected target variable. The target variable is typically a single variable of a primitive datatype, continuous or discrete, corresponding to the two most common machine learning tasks of regression and classification, respectively. However, there is a large number of practical domains with multiple target variables, such as image annotation (e.g., an image can depict sand, the sea, an umbrella and other objects), gene function prediction (where each gene can be annotated simultaneously with multiple functions from the gene ontology), predicting drug effects (each drug can have an effect on multiple medically relevant conditions) and document classification (e.g., a news report about Cristiano Ronaldo can be labelled with both sports and fashion). The common denominator of these domains is that they have multiple binary target variables and can thus be addressed with methods for multilabel classification (MLC). In MLC, an example can be labelled with a subset from a set of predefined labels^1,2,4^.

This paper introduces an online ontology-based catalogue of MLC datasets originating from various application domains. The catalogue extensively describes many MLC datasets with comprehensible meta-features and different data provenance information. The meta-features represent various measurable properties of the learning task^5^. Describing the MLC datasets with meta-features that capture the properties of the MLC task can allow for joint cross-domain investigation of the different MLC applications. The accumulation of meta-knowledge of this kind also allows the study of the task itself and improves the generalization performance.

More specifically, the benefits of such an MLC dataset catalogue containing a large set of meta-feature descriptors are threefold. First, the practitioners and non-machine-learning experts can better understand which MLC method to use for their specific use case or system. The use of the catalogue can reduce the user’s learning curve when selecting a method by a non-expert and promote adoption and trust in machine learning across existing and new domains. Second, it can allow experts to jointly reason about the properties of the learning task across different problems. Consequently, it can lead to a better understanding of the task and introduce novel MLC methods that can address the properties of the task under specific conditions. Third, the catalogue can be used as a benchmark environment to promote transparency when reporting results for a novel MLC method or cross-comparing different results.

The MLC catalogue contains descriptions of 89 MLC datasets in total. Each dataset is annotated with a set of different descriptors. Datasets can be seen as digital resources, and in that context, annotation is the process of attaching metadata about the concepts relevant to the resource being described. Our catalogue introduces a key novelty: all dataset descriptions are enhanced with semantic annotations (metadata) based on terms from ontologies and controlled vocabularies.

In the context of computer science, ontologies are “explicit formal specifications of the concepts and relations among them that can exist in a given domain”^6^. They provide means for knowledge and data representation that is semantically understandable and available in machine-processable form. Thus, ontologies have significant success in sharing a common understanding of information structure among people or software agents.

The inclusion of semantic annotations makes the catalogue complaint with contemporary standards of data management such as the FAIR (Findable, Accessible, Interoperable, and Reusable)^7^ and TRUST (Transparency, Responsibility, User focus, Sustainability, and Technology)^8^. The FAIR principles are a set of guiding principles that have been introduced to support and promote proper data management and stewardship. The TRUST principles go a level higher by focusing on data repositories and guiding their design and development.

The semantic annotations provide the means to develop several useful functionalities of the catalogue: (1) Semantic search over the corpus of annotated datasets; (2) Querying not only the asserted but also the implicitly encoded knowledge in the ontologies by using reasoners; and (3) Improved interoperability of the datasets with external data that follow the same conventions of data representation and management.

To allow users to access the catalogue and interact with it, we developed a user-friendly web-based system to inspect the pre-calculated MLC meta-features. Furthermore, the meta-descriptors are available for cross-comparison with similar datasets present in the catalogue via an interactive visualization engine. The publicly available datasets are also available for download for various tasks, such as evaluating novel MLC methods and different benchmark studies.

We publish the meta-dataset under the https://creativecommons.org/licenses/by/4.0/ licence. For each dataset in the catalogue, the semantic annotations can be downloaded in RDF (subject, predicate, object) triples. We provide a catalogue that the scientific community can utilize to improve the methods and promote the MLC task in various application areas. Finally, the semantic annotations (or the meta-dataset) are one of the main contributions of this work as they serve as a basis for the development of the data catalogue.

## Results

Our task in this paper was to design and implement an ontology-based catalogue of MLC datasets. This section highlights three significant contributions of our work: a set of MLC meta-feature descriptors, semantic annotations of MLC datasets that can be seen as a meta-dataset describing the MLC datasets, and a web-based system to explore and query the catalogue.

### Meta-features and the importance of the meta-knowledge

Meta-features describe the MLC datasets at the task level, as they encode knowledge about the task properties. As such, meta-features can be used as a base of an empirical study for the properties of the learning task or to guide practitioners to understand better the typical challenges encountered in solving problems like theirs.

MLC meta-features are divided into several groups: (1) Dataset specific meta-features, such as the number of attributes, data instances and labels; (2) Attribute specific meta-features, containing statistical and information-theoretic properties of the attributes; and (3) Label specific meta-features that describe the distribution of labels in the label space, as well as the relationship between the labels. A more elaborate taxonomy of meta-features is presented in the Methods section.

Combining the meta-feature description with the predictive performance of the MLC methods can shed light on the strengths and weaknesses of the different MLC methods on various datasets. It can also increase the corpus of meta-knowledge about the MLC task. Further, it allows for addressing different parts of the MLC pipeline, which is essential for machine learning practitioners. These include identifying the most suitable thresholding techniques, improving the re-sampling techniques, and augmenting the existing datasets, ultimately leading to a better generalization of the methods and providing better results.

In this context, our catalogue provides unified access to the MLC datasets and their properties, to researchers and practitioners, through a visual interface. The catalogue bridges the gap of various inconsistencies across the related repositories and works, providing a unique landscape of all of the MLC datasets and their properties. Its use can facilitate practitioners in addressing the MLC tasks, promote the adoption of MLC approaches among non-practitioners, and increase the trust in machine learning in other domains.

**Figure 1:**
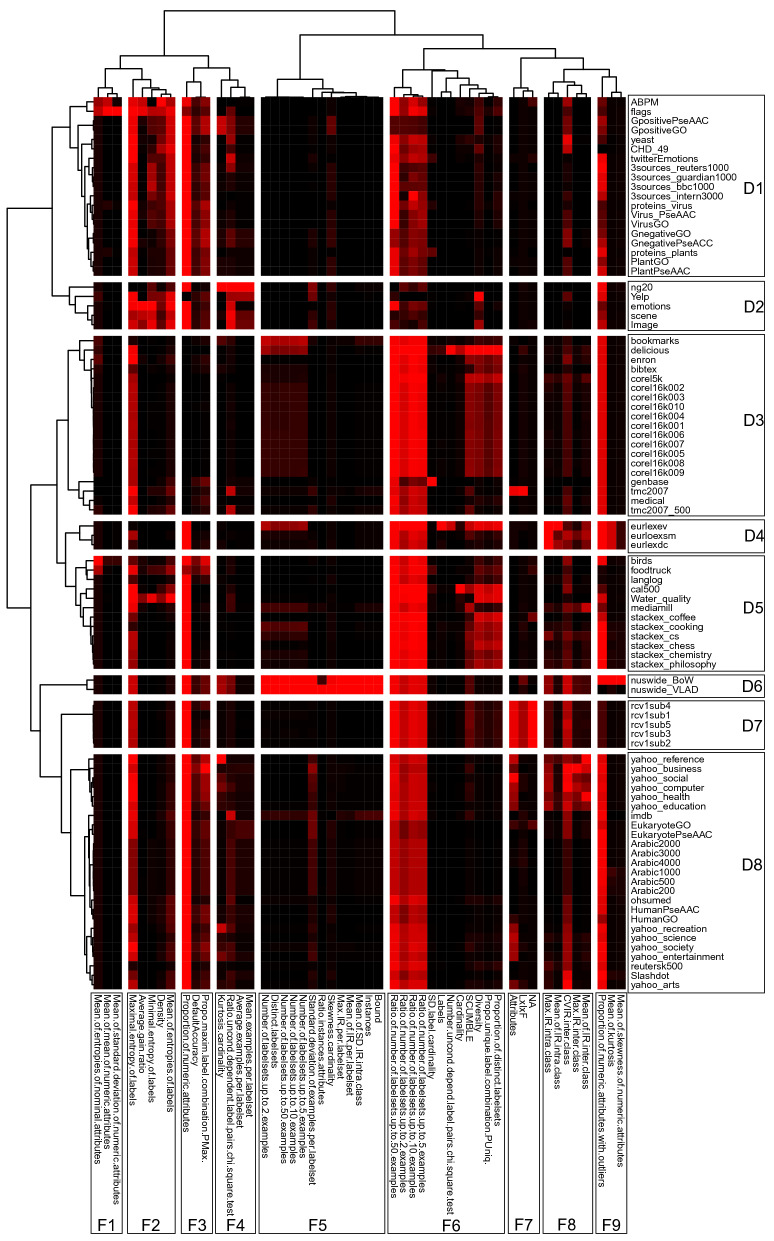
Heatmap of the calculated meta-features for all datasets in the catalogue. Rows represent the different datasets,while the columns represent the different meta-features. The values of meta-features are normalized to the scale 0–1 (black = 0 and red = 1). The colour of each square in the heatmap is the value of the appropriate meta-feature (column) for the appropriate dataset (row). The righthand side numbers denote dataset groups (marked with D), while bottom numbers denote meta-feature groups (marked with F). The heatmap was generated with R using the pheatmap package (version 3.6) (https://cran.r-project.org/web/packages/pheatmap/) and postprocessed by using Inkscape (https://inkscape.org/) version 1.1.2.

#### A landscape of MLC datasets

Here, we present a landscape of the MLC datasets included in the catalogue through the available meta features. This is essentially a use case showcasing the condensed meta-knowledge stored in our catalogue through a heatmap of the calculated meta-features for all datasets included in the catalogue (given in Fig. 1). We applied hierarchical agglomerative clustering with *Ward* linkage and *correlation* as a distance metric to both the dataset descriptions and meta-features. The obtained hierarchical clusters identify groups of datasets and meta-features that emerge as similar in the catalogue. The rows in the heatmap represent datasets, and the columns represent meta-features.

The obtained hierarchical clusters containing datasets or meta-features are represented in the top and the left-hand side of Fig. 1. These clusters group together the instances that are closer to each other according to the specified distance metric – the more similar datasets are grouped into the same cluster and conversely the more similar meta-features are grouped into the same cluster. Determining the optimal cutting point in the hierarchical clustering is not a straight-forward task – typically, domain expert knowledge is required to perform the selection^3^. This is what we did in our case: based on our knowledge of the domain of MLC datasets and meta features, we decided on the granularity of the clusters and determined the cutting point in the hierarchical clusters.

In general, if we observe Fig. 1 along the dimension of datasets (rows), we can identify eight groups of datasets (the groups of datasets are marked with label D). In comparison, if we observe along the dimension of meta-features (columns), we can identify nine meta-features groups (the meta-features are marked with F). Some of the dataset groups are rather homogenous. For example, group D5 contains two datasets, NuswideBow and NuswideVlad, that originate from the image domain. The datasets share the same target space and have the same number of instances but have different feature spaces. These small differences result in clustering together of these two datasets, which are relatively far from the others (as seen by the height of the connecting dendrogram point).

When we observe group D8, we can see another sizeable homogenous group which can be labelled as the *Reuters* datasets, which contain news articles and are predominantly textual. They are different from the other groups by having significant complexity in terms of the product between the number of instances, labels and features (group F7). These datasets are characterized by a smaller number of instances per labelset (as seen by the high values of the meta-features from group F6) and a large imbalance between the labels (seen in group F8). Within this group, there are two large subgroups of text datasets: *yahoo* and *Arabic*.

When comparing the dataset groups, the most interesting difference is observed between the groups D1 and D8. Compared to the latter, the former is characterized by balanced distributions of the samples per labelset. As shown in Bogatinovski et al.^9^, this feature group is very important for the appropriate algorithm selection when dealing with multilabel classification problems.

The analysis of the meta-feature groups reveals several interesting observations. For example, the F2 meta-feature group is characterized with higher entropy as opposed to the remaining groups. The F5 meta-feature group is discriminative for a large number of label sets. It is the most discriminative meta-feature group for the *Nuswide* datasets against others (group D6). As previously discussed, the complexity of the dataset group D7 is characterized by the high complexity in terms of features, instances and labels. A large number of numeric features characterizes the D1 dataset group, as opposed to group D3, which is characterized by a discrete descriptive space. Therefore, the meta-features depict the whole landscape of the datasets and the different groups. The detailed description of the whole meta-feature landscape is essential for many reasons, such as benchmarking data selection and studying the properties of the task.

To summarize, the meta-features are successful in describing datasets with different complexities. If combined with the predictive performance of the methods, one can obtain valuable insights and transfer the knowledge about what methods work better in what domains.

### Semantic annotation of MLC datasets

The enrichment of the MLC datasets with semantic metadata is one of the main prerequisites for the creation of a catalogue governed by the FAIR principles^7^. For semantic annotation of datasets, we have designed an ontology-based schema that enables the description of multiple aspects of MLC datasets. The schema is an adaptation of a more general annotation schema that covers a broader range of machine learning tasks presented in Kostovska et al.^10^. We can broadly categorize the semantic annotations into two groups: (1) Annotations of datasets with provenance information and (2) Annotations that capture relevant machine learning characteristics of the datasets.

Provenance information refers to the kind of information that describes the origin of a resource (in our case, resources are datasets), i.e., who created the resource, when was it published, and what is its usage license. For semantic description of provenance information, we have chosen the Schema.org vocabulary, a shared collection of schemas widely used for providing structured data on the Web. Specifically, for annotation of MLC datasets, we are using the Dataset schema^11^ from Schema.org^12^. It provides a list of properties that can be used for annotation, such as name, description, identifier, citation, and license, among others. It should be noted that when we semantically annotate a dataset, we usually use a subset of these properties as the complete provenance information is not always provided.

From an ML perspective, we find different types of annotations relevant and beneficial, such as dataset specification, learning task, datatypes, and meta-features. To enable annotation of ML specific information, we have combined ontological concepts from two external ontologies, i.e., the ontology of core data mining entities (OntoDM-core)^13^, and the ontology of datatypes (OntoDT)^14^. Furthermore, we have added concepts relevant to our domain, such as concepts that semantically define the meta-features, and have proposed an MLC dataset annotation schema.

#### Example annotation of a MLC dataset

**Figure 2:**
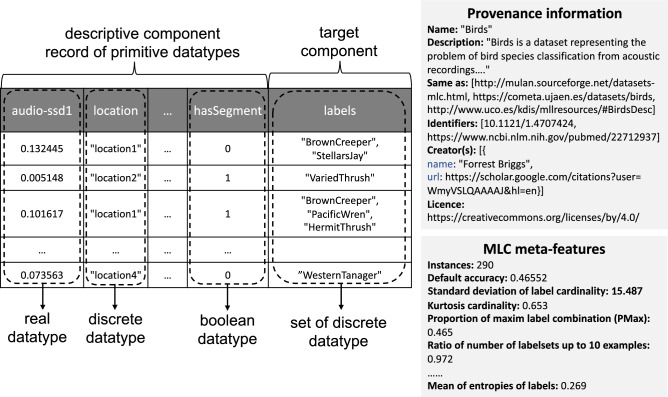
Semantic annotation of the Birds dataset^15^. We depict the dataset in tabular format on the left-hand side, where we also show the annotations of the different datatypes present in the dataset. In addition, we present the annotations of the available provenance information and a list of calculated MLC meta-features on the right-hand side.

In Fig. 2, we present an example of a semantic annotation of the Birds dataset^15^. On the left-hand side, we show an outline of the dataset in tabular format and show the annotations of the different datatypes present in the dataset (e.g., the annotations of the descriptive attributes and the annotations of the labels). On the right-hand side, we present an outline of the annotations of the available provenance information such as name, description, URL and licence and a list of several calculated MLC meta-features.

### Exploring and querying the MLC dataset catalogue

We have developed a web-based system with a simple user interface (UI) to facilitate the accessibility of the developed catalogue of MLC datasets and the generated semantic annotations. Users can easily search the catalogue for MLC datasets and interactively explore their characteristics. The system can be accessed via the URL http://semantichub.ijs.si/MLCdatasets.

**Figure 3:**
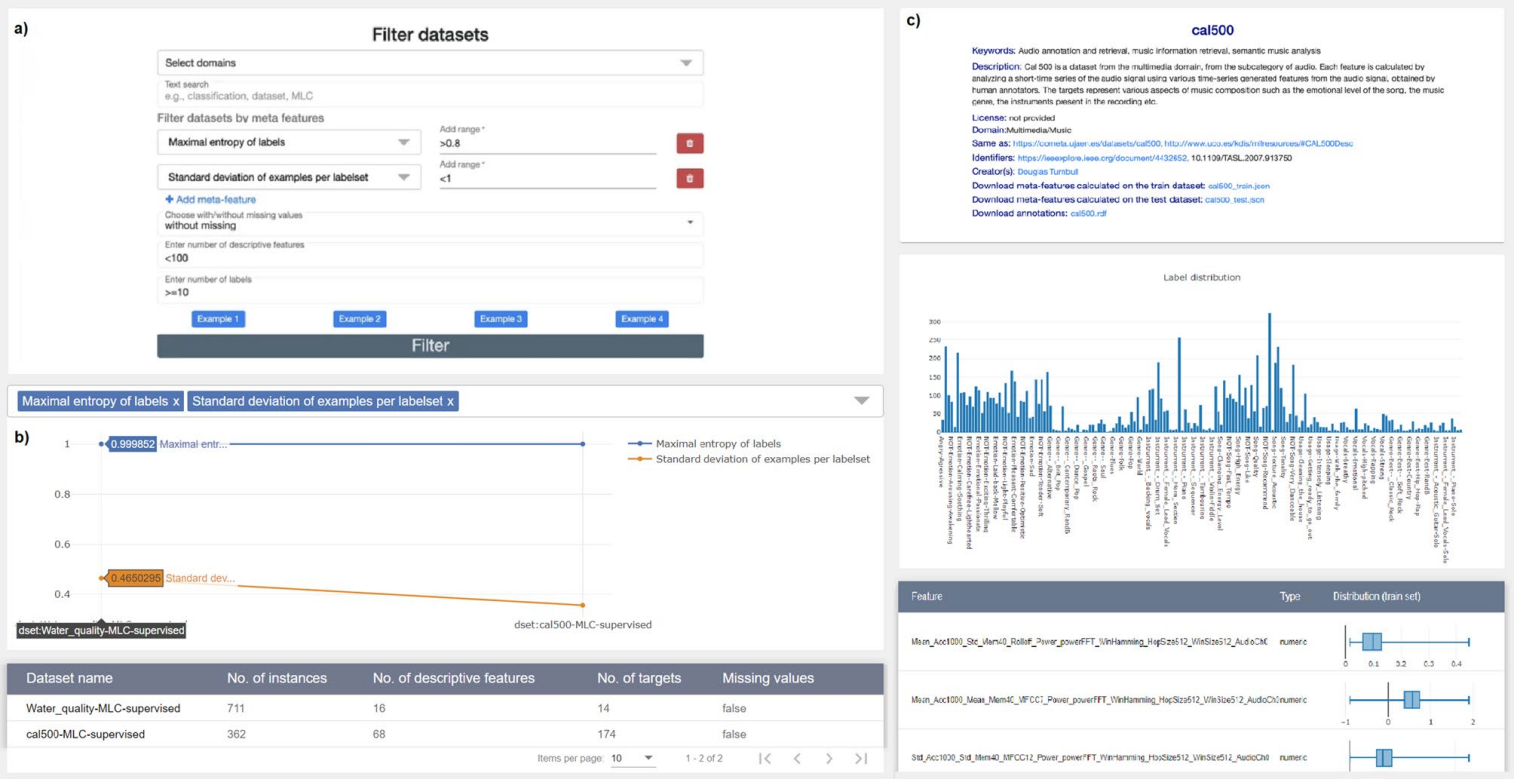
A screenshot of the multiple views developed within the GUI of the web-based system. (**a**) A filter for querying datasets according to a set of parameters. (**b**) Table with the results from the filter query and a scatter plot for visualizing the MLC meta-features. (**c**) A view of an individual dataset. It includes a panel for provenance information and links for downloading the raw dataset and the generated semantic annotations, a label frequency bar chart, and a table with the descriptive attributes present in the dataset and their distributions (box plots).

The UI consists of three components with different functionalities: (1) Browse datasets, (2) Filter datasets, and (3) Meta-features. The “Browse datasets” functionality allows users to browse the complete meta-dataset. The “Filter datasets” functionality allows users to search for specific MLC datasets that satisfy a set of user criteria. Finally, the “Meta-features” functionality lists all the meta-features with their descriptions and equations in LaTex format. It gives the user opportunity to explore all datasets for the chosen meta-feature.

Figure 3 depicts the main view components of the UI for filtering datasets according to user-specified criteria. First, a filter page provides end-users with an easy-to-use search tool to graphically parameterize predefined types of queries. The users can specify different search parameters. These include the application domains of the datasets, text search of the datasets’ provenance information, the number of descriptive and/or target features the dataset has, the value ranges of the different MLC meta-features, and whether the dataset has missing values (with missing/without missing). These search parameters are specified via input fields or predefined drop-down menus that contain ontology terms. The semantic queries (expressed in SPARQL) are automatically generated in the background (see Fig. 3a) and are transparent to the user of the corresponding system.

The result of the executed query is presented in a results page view (see Fig. 3b). This view contains a table with all the datasets resulting from the previously defined query. Additionally, an interactive plot that visualizes the distribution of MLC meta-features is shown. MLC datasets that satisfy the user-defined conditions are plotted on the x-axis, and the meta-features are plotted on the y-axis. Users can interactively add or delete meta-features from the scatter plot.

When the user clicks on a particular dataset from the table, another page view is rendered (see Fig. 3c). Here, we have specific information for the chosen dataset, such as the available provenance information and links to access the datasets, the semantic annotations, and the complete list of meta-features. The user can also download the complete set of annotations in RDF format. We also provide a histogram that depicts the label distribution of the dataset and a table that shows each feature in the dataset (name, type, and distribution). In addition, box-plots and bar-plots display the distribution of numerical and nominal features, respectively.

## Discussion

While there exist several repositories and collections of MLC datasets^16–20^, the catalogue we provide is the most extensive one. To the best of our knowledge, our catalogue links to the largest number of publicly available MLC datasets, 89 datasets to date, and hosts semantic annotations of those datasets, including the calculated meta-features. Another feature of our catalogue as compared to other MLC repositories, is the interactive nature of the catalogue supported by the underlying web-based system. More specifically, the web-based system allows users to interactively inspect all of the available datasets based on the provided semantic annotations. Moreover, most of the currently available repositories list a subset of the meta-features we provide in the form of tables, making the joint inspection of the differences and similarities between datasets more difficult. Our web-based system provides a more accessible, visual way of inspecting the datasets and their meta-features.

We also provide the links to the train and test splits of the datasets as used in one of the most extensive studies of MLC methods^21^. Providing information about the train/test splits is especially important for benchmarking and reproducibility of computational experiments. More specifically, this helps to facilitate the comparative evaluation of novel methods on many datasets at once, without the need to train novel models by competing methods. Furthermore, the objectivity of this approach allows for improved comparison of the newly introduced methods with their competitors that represent the actual state in the MLC area at the current time. Finally, the embedded knowledge in the catalogue encourages reusability among both researchers and machine learning practitioners.

The catalogue of MLC datasets makes a special effort to calculate the MLC meta-features. Having all the calculated meta-features in one place allows the experts to jointly observe the properties of the learning task across different problems. Moreover, it can answer many task-specific questions, e.g., which MLC methods perform best in what situation, how the properties of the learning task influence the behaviour of the MLC methods, and others. A significant additional opportunity exists to combine several suitable methods on similar problems and improve the generalization and the time needed to build a model for the novel problem.

To conclude, the main contribution of our catalogue is the use of a semantic layer for representing standardized, formal descriptions of datasets through the application of formal ontologies. The rich semantic annotations provide the catalogue with advanced querying capabilities that employ the reasoning power of ontologies. Furthermore, the explicit inclusion of semantics further broadens the range of applications of the available datasets, as this helps practitioners better understand, reuse and augment the data automatically. Finally, the uniqueness we provide along various dimensions makes our catalogue the go-to source of datasets for future benchmarking and evaluation of MLC methods.

## Methods

In this section, we briefly describe the methodology employed in constructing the MLC dataset catalogue. First, we describe the task of multilabel classification and the meta-feature descriptors that can be calculated for the MLC datasets. Next, we present the design of the semantic annotation schema used for annotating the MLC datasets. Finally, we focus on the design and implementation of the web-based system for exploring and querying the MLC dataset catalogue.

### The task of multilabel classification

MLC is a machine learning task where the goal is to predict the subset (out of a predefined set) of labels that are relevant for a given data example^1,2,22,23^. By doing so, for each data example, two sets of labels are defined: relevant and non-relevant labels-such a modelling approach results in improved predictive performance and widespread adoption of the MLC task. We have witnessed the broad use of the MLC methods in diverse interdisciplinary applications ranging from areas in biology, bioinformatics, chemistry, medicine, video, audio, images, text, and the number of applications is constantly increasing^21,24^.

The different application domains introduce interesting properties relevant to solving the MLC task. For example, for problems in bioinformatics of gene sub-cellular localization, the number of associated labels for each instance is smaller from problems from the textual domain, where usually an object is associated with many different labels. Moreover, the later domain usually assigns the textual object with multiple categories, e.g., politics, economy, national, instead of the former, where the location of a gene in a cell is more constrained. These different properties can lead to a preference for specific learning methods over others depending on their ability to utilize the available information. Other unique, essential properties of the MLC task are the imbalance of labels and labels dependency.

### Meta-features

**Figure 4:**
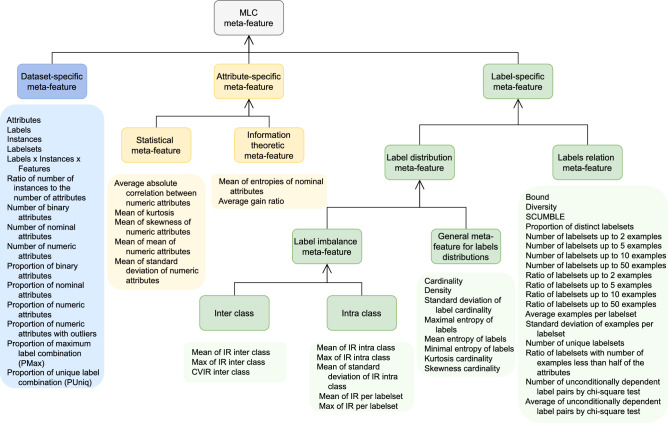
The taxonomy of meta-features for describing multilabel classification (MLC) data sets.

In order to characterize the MLC datasets, we calculate various meta-features. All meta-features are calculated using two Java-based libraries, i.e., MLDA^25^, and MULAN^19^, that implement the meta-features. In Fig. 4, we show the taxonomy of meta-features (MF) used to describe the MLC datasets in our catalogue. They are separated into three major groups based on the part of the datasets they describe (general dataset characteristics, attributes and labels).

*Dataset-specific meta-features* describe the datasets from the perspective of general statistics. This group of MFs includes the number of instances, number of labels, number of features, their type and various ratios between them. They provide a general landscape for the dataset and its complexity according to the three dimensions: instances, attributes and labels.

*Attribute-specific meta-features* provide a detailed insight into the properties of the attributes by calculating various properties of the numerical and nominal attributes. These features are grouped into two subgroups: statistical and information-theoretic meta-features.

*Label-specific meta-features* are concerned with describing the label space of MLC datasets. They are split into two subgroups based on the two essential properties of the MLC task: the imbalance across labels and the high dimensionality of the label space. The label sets distribution group has various properties describing the distributions within the labels and the samples. Based on the approach taken in calculating the features, they can be grouped into further subgroups as depicted in Fig. 4. The relationships among labels include various properties, such as the maximal variation between the labels within the label sets for all of the examples, the number of label sets, statistical tests of dependence and others.

### Semantic annotation schema design and implementation

To design the semantic annotation schema, we followed the OBO Foundry principles^26^. With this, we ensure interoperability with existing standards (e.g., unique identifier space, using a common formal language for ontology development, and employing upper-level ontologies, such as the BFO ontology^27^). Also, we reused classes from already existing ontologies from the domain of machine learning and data mining, i.e., OntoDM-core and OntoDT.

**OntoDM-core**^13^ is an ontology of core data mining entities. OntoDM-core provides a framework for describing the key DM entities, i.e., dataset, DM task, generalizations, DM algorithms, implementations of algorithms, DM software. The entities are described in a three-layered ontological structure which includes a specification, an implementation, and an application layer, allowing flexibility in ontology use. OntoDM-core also defines taxonomies, such as taxonomies of datasets, data mining tasks, and data mining algorithms.

**OntoDT**^14^ is a generic ontology for representing knowledge about datatypes. It was initially designed for generating descriptors of datatypes for data from the domain of data mining and using them to define the data mining tasks and the set of applicable algorithms. However, its usage is not restricted, and it can be applied to a variety of domains^28^. The central class in the ontology is the *datatype* class. It defines the type of data, the set of distinct values of the data, datatype properties (order, numericalness, cardinality, equality, and boundedness), and the set of operations that can be performed on the data.

**Figure 5:**
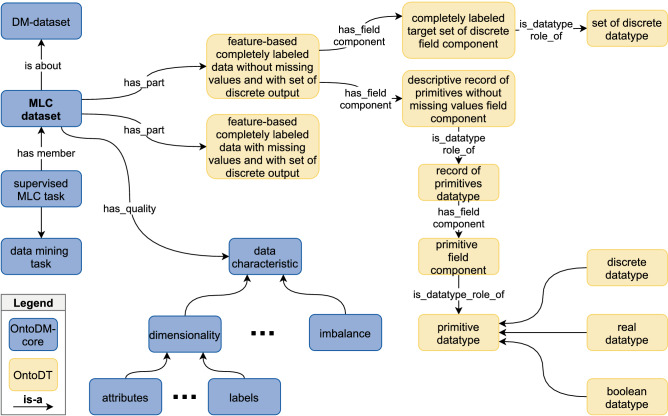
ML-specific annotation schema for MLC datasets based on the OntoDM-core^13^ and OntoDT^14^ ontologies. The schema allows annotation of the different datatypes appearing in the datasets, specification of the data mining task, and representing MLC-specific meta-features as data characteristics, which are connected with the MLC dataset via the has_quality property.

Figure 5 depicts the high-level view of the proposed annotation schema. First, the MLC datasets are represented as instances of the *MLC dataset* class, which in OntoDM-core is modeled as a dataset specification of feature-based data^13^. In order to explicitly encode the learning task, which in our case is MLC, we connect the *MLC dataset* class and the *supervised MLC task* via the has-part relation.

To represent the datatypes, we reuse classes from the OntoDT ontology. For example, in the case when the data examples do not contain missing values for the descriptive features, we reuse the *feature-based completely labelled dataset without missing values and with a set of discrete output* class. Each data example is composed of two components, i.e., a descriptive component that contains the descriptive features and a target component for the target labels. For each of the components, there is a corresponding datatype. Then, the datatypes are refined until a primitive (boolean, discrete, real) datatype is reached. A more detailed description of the taxonomy of datatypes, their use in the context of machine learning and their representation can be found in Panov et al.^14^.

Finally, in order to provide the annotation of the MLC dataset meta-features, we extended the *OntoDM-core* ontology. We also reused the taxonomy of meta-features proposed by Moyano et al.^25^. To this end, each item in the taxonomy was represented with a corresponding class in the *OntoDM-core* ontology as a subclass of the *data characteristic* class (see Fig. 5). Accordingly, datasets can be annotated with additional information about the meta-features, their concrete values, and the time of calculating the meta-feature expressed in milliseconds.

### Design and implementation of the web-based system

In the background, the MLC dataset catalogue is supported by a system that automatically generates the semantic annotations and facilitates the execution of semantic queries for easy access to the datasets. Here, we describe the design and implementation of the system and its components and the workflow for annotation and querying of datasets, powered by the extensive use of Semantic Web technologies.

**Figure 6:**
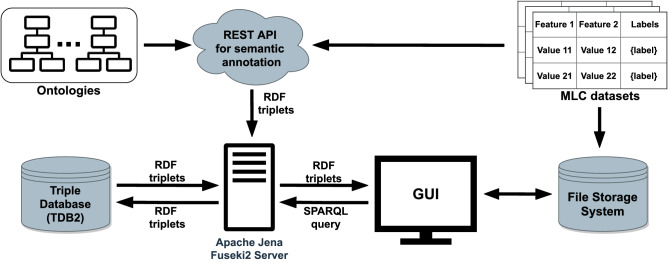
Client-server architecture of the web-based system behind the MLC dataset catalogue. The system comprises of several components, i.e., REST API for semantic annotation of datasets; a triple database, Apache Jena TDB2, for storage of the annotations in RDF format; an FTP file server to store the raw datasets in ARFF format; an Apache Jena Fuseki2 server that handles the semantic query requests; and a GUI that supports intuitive and interactive exploration of the datasets and the accompanying knowledge base.

Figure 6 depicts the general client-server architecture of the web-based system behind the MLC dataset catalogue. The system comprises several components, i.e., graphical user interface (GUI), REST API for semantic annotation, file storage system, triple store database for storage of the annotations, and a server, which serves as an endpoint for querying the semantic knowledge base.

For the development of the REST API for semantic annotation of MLC datasets, we used the Java Spring framework. The API expects as input an MLC dataset in the Weka’s ARFF format^29^, a JSON-LD file that specifies the available provenance information and the ontology-based annotation schema. The annotations are then generated via the Apache Jena library as sets of RDF (subject, predicate, object) triples.

Once the annotation process is completed, the produced annotations are sent to the Apache Jena Fuseki2 server^30^. The Fuseki server further handles the request by uploading the RDF triples to the Apache Jena TDB2^31^, which can be accessed via SPARQL^32^ queries. Also, alongside the annotations, we store the inferred versions of ontologies to speed up the execution of the queries that require reasoning. The inference is made using the OWL Micro reasoner^33^. Finally, the raw datasets are stored on a file server and can be retrieved on request through the FTP protocol.

At the client side, we have a GUI that hides the complexity of the ontology-based annotations and the annotation schemes from the end-users and implements the functionality of querying the knowledge base without any proficiency in writing SPARQL queries on the part of the user. Instead, the user formulates the query by providing information in the input fields or by clicking predefined drop-down menus that contain labels of the ontology classes used for annotation. The SPARQL queries are generated in the background and propagated to the Apache Jena Fuseki2 server based on the input. The GUI was implemented using the Angular 7.0 framework, the latest stable version of Angular at the time of development^34^.

## Data Availability

This work contributes meta-datasets describing MLC datasets in a joint meta-space. We generate a separate meta-dataset for each of the MLC datasets in the catalogue. The meta-datasets are publicly available at http://semantichub.ijs.si/MLCdatasets through the web-based interface, published under the https://creativecommons.org/licenses/by/4.0/ license and can be downloaded in RDF format. Also, the MLC datasets that are open and publicly available can be downloaded directly from the web catalogue in ARFF format. The calculated MLC meta-features appear in the meta-dataset but are also available for download in JSON format. All associated code is hosted on GitHub (https://github.com/KostovskaAna/MLC-data-catalog).
